# Road risk-perception and pedestrian injuries among students at Ain Shams University, Cairo, Egypt

**DOI:** 10.5249/jivr.v4i2.112

**Published:** 2012-07

**Authors:** Jehan M. Ibrahim, Hannah Day, Jon Mark Hirshon, Maged El-Setouhy

**Affiliations:** ^*a*^Faculty of Medicine, Ain Shams University, Cairo, Egypt.; ^*b*^Department of Epidemiology and Preventive Medicine, University of Maryland School of Medicine, Baltimore,USA.; ^*c*^Department of Emergency Medicine, University of Maryland School of Medicine, Baltimore, USA.

## Abstract

**Background:**

Road traffic injuries (RTIs) constitute 45% of injury mortality in Egypt; 75% of these injuries are pedestrians related. Traditionally, research on road traffic safety has focused on the traffic environment and the vehicles. However, little attention has been given to road risky behaviors and perceptions of road safety by pedestrians as risk factors associated with high pedestrian injury rates. This study aimed to examine the relationship between road risk- perception, specific road behaviors, and self-reported pedestrian injuries among university students in Cairo, Egypt.

**Methods:**

A cross sectional survey was conducted among university students aged 18 to 24 years old at Ain Shams University in Cairo. Questions covered socio-demographic variables, injury episodes, road behaviors, road risk-perceptions, attitudes towards injury prevention, and road safety education.

**Results:**

The survey was completed by 1,324 students. Two hundred ninety (21.9%) of the participants suffered from pedestrian injury during the past 6 months; of these, 28.9% were admitted to hospital or clinic as a result of the injury, 39.3% were unable to go to university or leave home because of the injury for a period ranging from one day up to one week. Participants were more likely to suffer from pedestrian injury when they did not always "look both ways to cross the road", whereas always "waiting for a green light" was protective. Students who "perceived it safe to cross the road from any point" or "did not perceive it to be safer to cross at a zebra crossing" were less likely to "look both ways" before crossing the road. Similarly, there was a positive association between road safety education and participants’ road crossing behaviors.

**Conclusion:**

Inappropriate youths' road behaviors were significantly associated with pedestrian injury. There was also a positive association between road risk perception and road behaviors. This suggests that a behavioral approach together with modification of the traffic environment (such as provision of crossing signals) might be effective in preventing the occurrence of pedestrian injury.

## Introduction

Road traffic injuries (RTIs) constitute 45% of injury mortality in Egypt; 75% of these injuries are pedestrians related.^1^Traditionally, research on RTI has focused on the traffic environment and the vehicles.^2^However, little attention has been given to road risky behaviors and perceptions of road safety by pedestrians as risk factors associated with high pedestrian injury rates.

Pedestrians, particularly adolescents, often show risky behaviors when crossing roads, such as not checking for oncoming traffic.^3^Disobeying pedestrian traffic rules has clearly been associated with pedestrian injuries leading to substantial morbidity and mortality.^4^Approximately 90% of disability adjusted life years lost, worldwide, due to RTIs occurs in low and middle income countries.^5^In these countries, often, road crossing is not an easy or safe task, since roads commonly have limited pedestrian facilities, therefore, forcing individuals to mix with heavy traffic traveling at different speeds. Moreover, RTIs are rapidly increasing in low and middle income countries due to rapid motorization, poor enforcement of traffic safety regulations, and inadequate health infrastructure to provide adequate treatment for those injured in the roads^5^

Egypt, as a middle income country, has a substantial number of injuries and deaths related to pedestrians being struck by vehicles. Cairo is well-known for its road traffic and crowded sidewalks and streets, which exacerbate the problem. An average 77% of the sidewalks are blocked by encroachments which force pedestrians to step on the road resulting in vehicle swerving.^5^An average 33% of the streets are blocked by illegally parked vehicles.^5^

Despite its unquestionable impact on Egypt, limited attention has been paid to pedestrian injuries as a major public health problem.^6^One of the reasons for lack of attention is the deficiency of reliable and valid information about these types of injuries. This knowledge deficit hides the extent of the problem from policy makers. As it is not enough to know the magnitude of the pedestrian injury problems among youth (which is an impressionable time of life when many adult habits are developed), we explored their road risk perception. We also need to investigate the common road behaviors that could precipitate pedestrian injuries.

Safety education for youth could be an important factor in preventing road injuries as it has been shown that driver’s and pedestrian’s road safety knowledge, both, are poor and inadequate in low and middle income countries.^7^

 Efforts to modify pedestrians' behavior and the environment they negotiate should be considered in order to reduce pedestrian injuries and/or deaths. In this study we examined the relationship between road risk-perception, specific road behaviors, and self-reported pedestrian injuries among university students in Cairo, Egypt.

## Methods

**Study design and Population**

A descriptive cross-sectional survey. Participants were university students from different faculties/colleges, between 18 and 24 years and of both sexes.

**Sampling**

There are 16 faculties/colleges at Ain Shams University. First, we categorized them into two main categories: seven scientific and nine academic faculties. We did this to ensure inclusion of all types of faculties with different academic characteristics.

Next, we randomly selected two scientific (medicine, engineering) and 2 academic (commerce, literature) faculties. University students from 1st to 4th year in college were included from both scientific and academic faculties (total number of registered students at these faculties are 12,650 and 32,200, respectively).

Accordingly, a total sample size of at least 1020 students was calculated, based on the 24% and 22% of pedestrians, 20 years old and older, reported being injured in the past year while walking in Madi and Nasr City districts, Cairo governorate.^6^

Next, a stratified random sample was obtained proportional to the total number of students registered in each type of faculties. Hence, 287 and 733 students were surveyed from academic and scientific faculties, respectively.

Finally, a systematic random sample was drawn from the students' attendance lists in which the first student ID number was randomly selected and then every 40 ID number (according to the calculated skip interval). The students were approached and invited to participate in a self-administered survey at their classrooms. During the administration of the survey, study supervisors were present to answer students’ questions and ensure full completion of the survey by the participants.

An 80% power, 5% significance level and 95% confidence interval (CI) were used in the sample size calculation. A 10% drop out rate (due to incomplete questionnaires or non response) was taken into consideration during sample size calculation using Epi Info 6, software. 

**Ethical Considerations**

This research was reviewed and approved by the Research Ethics Committee of Ain Shams University, Cairo, Egypt.

**Study Questionnaire**

The design of the questionnaire ensured that it was sufficiently clear for self-completion. The phrasing of the questions was carefully considered to avoid any ambiguity and potential misinterpretation. A major factor in obtaining cooperation to respond to questionnaires is the time it takes to complete.^8^ It was, therefore, imperative that questions were kept to a minimum but still generated the data needed. The questionnaire covered injury episodes, road behaviors, road risk perception and attitudes towards injury prevention, road safety education, and socio-demographic variables. This study included injury episodes that occurred in the 6 months preceding the survey. We used a short recall period (3-6 months) as recommended by previous studies, ^9^which found that longer recall periods underestimate injury rates compared with shorter recall periods. Pedestrian injury was defined as an injury incurred as a result of being hit by any type of vehicle on the road during activities such as crossing, walking, or playing, regardless of where the collision took place.

Injury episodes were investigated by whether the injury event required admission to hospital or clinic; limited college participation; the duration of limitation, and if there is any residual disability as a result of this injury.

Attitudes towards RTI prevention were measured by whether RTIs could be prevented. The responses were indicated on a five-point Likert scale ranging from 1 (not at all) to 5 (always). These responses were recorded dichotomously into "no" which included the response "not at all" and "yes" which included responses from "a little bit" to "always"

Road behaviors were evaluated by the frequency of looking both ways before crossing, waiting for a green signal before crossing the road, walking on the road, and walking down the sidewalk. The participants indicated the frequency of performing these behaviors on a five-point Likert scale ranging from 1 (never) to 5 (always). These responses were recorded dichotomously: "always" for road crossing and "never" for road walking behaviors, versus all other responses. This is because we defined "safe behavior" for road crossing as always looking both ways or always waiting for a green signal before crossing the road, and for road walking as never walk in the road.

Participants’ road risk-perception was measured by asking how safe did participants think it was to cross the road from any point, in relatively little traffic, at a zebra crossing, and to play on the road. The responses were indicated on a five-point Likert scale ranging from 1 (not at all) to 5 (always). These responses were recorded dichotomously into "yes" which included responses from "moderately" to "always", and "no" that included response of "not at all" or "a little bit". 

Road safety education was measured by frequency of safe road crossing behaviors discussion with parents, lecturers and friends. Participants were categorized as frequent education if they responded “always" or “very frequently", and infrequent education if they responded "not at all", "a little bit" or "moderately". 

**Data Management and Analysis**

Data was stored and analyzed using SPSS software version 13 and SAS JMP 5.0.1.2. Chi-square test was used forinitial analyses to assess differences between groups.

Main variables we aimed to explore were: Injury episodes, attitudes towards injuries, road behaviors, road risk-perception, and road safety education.

**Injuries and Attitudes towards Injuries**

Risk factor analysis was conducted comparing respondents who had had an injury in the past six months to those who had not (dependent variable). All variables that were potentially significant (α=0.1) in the bivariate analyses were included in the initial (full) multivariable logistic regression model. These significant factors were: ever play in sidewalk, ever play in the road, infrequent parental education on safe road crossing and attitude towards RTI prevention. In the multivariable analysis variables not significantly associated (α =0.05) with the outcome were removed from the model. The resulting multivariable logistic regression model was considered the final model and was used to calculate odds ratios (OR) and 95% CI for the remaining risk factors.

All of the ORs presented are those for a particular factor controlling for all other significant variables (listed earlier) in the model.

**Road Behaviors**

Road safety behavior analyses were adjusted for gender since this is an important covariate as indicated in the literature.^10^According to the sexual selection theory males tend to behave in ways that are more risky than females. Risk factor analysis was completed for a summary score of road behaviors. The score gave 1 point for each of four risk behaviors (1.not always looking both ways before crossing the street, 2 .not always waiting for green signal to cross, 3 .ever playing in the road or 4. ever playing in the sidewalk). Analyses were completed examining those who scored four i.e. having four risk behaviors, compared to those who scored one (i.e. having one risk behavior), three compared to one, and two compared to one. There was no one, among participants, who scored zero (i.e. there isn't any participant without any road risk behavior). All variables with a P-value ≤ 0.10 were entered into the full model for each comparison (4 vs. 1, 3 vs. 1, 2vs. 1). Only variables that were significant at p=0.01 (to correct for multiple comparisons) were left in the final models.

**Road Risk Perception**

Risk factor analysis was completed for a summary score of road perceptions. A score was developed based upon giving one point for each negative road risk perception. One point was given if the participant thought it was safe to cross at any point in the road, another point if the participant thought it was safe to cross in relatively little traffic, another point if the participant thought it was safe to play in the road, and another point if the participant did not think it was safe to cross at a zebra crossing. A bivariate analysis was performed examining the association between those who had scored a four i.e. had four negative road risk-perceptions, compared to those who had scored a zero i.e. had no negative road risk- perception. Similar tests were performed for those who had scored three compared to zero, and two compared to zero. All variables with a P-value < 0.10 in the bivariate results were entered into the full model for each comparison. Only variables that were significant at p=0.01 (to correct for multiple comparisons) were left in the final models.

## Results

**Demographic Characteristics / Injury Episode Description**

We received 1324 fully completed questionnaires out of 1500 distributed (response rate 88.3%). The participants’ mean age was 19.8 + 1.4, with 58.1% females. Among participants, 92.4% lived in urban areas. 76.5% lived more than 10 minutes away from university and 86.6% had to cross one or more roads on their way to university. Two hundred ninety (21.9%) of the participants suffered from pedestrian injury during the past 6 months; of these, 28.9% were admitted to hospital or clinic as a result of the injury, 39.3% were unable to go to university or leave home because of the injury for a period ranging from one day up to one week. Only 21.4% of participants who sustained a pedestrian injury had some kind of residual disability resulting from this injury.

Socio-demographic factors associated with pedestrian injury included residing in rural areas, living more than 10 minutes from the university, crossing one or more roads to the university, and having a mother who was not employed outside the home (Table 1).

**Table:** Table 1: **Variables associated with self-reported pedestrian injuries **

Variables	Total (%)^**^ n = 1324	Number injured (%)^♦^ n = 290	OR^♣^	95% CI	χ^2^(P value)
Gender					
- Male	555 (41.9)	120 (21.6)	0.98	0.78 - 1.33	0.02 (0.46)
- Female	769 (58.1)	170 (22.2)			
Residence					
- Rural	100 (7.6)	34 (34)	2.15	1.32- 3.49	9.39 (0.002)
- Urban	1224 (92.4)	256 (20.9)			
Father's education					
- Illiterate	45 (3.4)	13 (28.9)	1.34	0.84 – 2.14	1.36 (0.17)
- Literate	1279 (96.6)	277 (21.7)			
Mother's employment					
- Housewife	730 (55.1)	179 (24.5)	1.32	1.10 - 1.64	6.91 (0.03)
- some employment	594 (44.9)	111 (18.7)			
Distance to university					
- More than 10 minutes	1013 (76.5)	232 (22.9)	1.2	0.96 - 1.62	2.92 (0.03)
- Up to 10 minutes	311 (23.5)	58 (18.6)			
Road crossing to university					
- One or more crossing	1146 (86.6)	272 (23.7)	2.93	1.72 - 4.96	16.54 (0.00)
- No crossing	178 (13.4)	18(10.1)			
Looking both ways before crossing road					
- Not always^*^	427 (32.3)	125 (29.3)	2.00	1.50- 2.67	19.21 (0.00)
- Always!	897 (67.7)	165 (18.4)			
Wait for green signals before crossing road					
- Not always^*^	1101 (83.2)	230 (20.9)	0.77	0.61- 1.00	4.10 (0.02)
- Always	223 (16.8)	60 (26.9)			
Walking/playing in road					
- Never	170 (12.8)	39 (22.9)	1.06	0.79- 1.42	0.14 (0.39)
- Ever^#^	1154 (87.2)	251 (21.8)			
Walk/play on sidewalk					
- Never	47 (3.5)	9 (19.1)	0.87	0.48- 1.61	0.21 (0.39)
- Ever^#^	1277 (96.5)	281 (22.0)			
Attitude to RTI prevention - RTI can be prevented					
Yes^!^	283 (21.4)	211(74.6)	1.52	1.13- 2.06	7.61 (0.004)
No^ε^	1041 (78.6)	79(7.6)			

* The category "not always" included responses from "never" to "frequently". # The category "ever" included responses from "rarely" to "always". ε The category "no" included the response "not at all". ! The category "yes" included responses from "a little bit" to "always" ** The percentages are column % ♦ The percentages are row % ♣ All of the ORs presented are those for a particular factor controlling for all other significant variables in the model.

**Injury Associated Risk Factors**

Residents of rural areas had odds of injury that were 2.15 (95% CI = 1.32-3.49, p-value < 0.01) times higher than urban residents. Having one or more road crossings had odds nearly three times higher (OR = 2.93, 95% CI = 1.72-4.96, p-value < 0.01) of having an injury than those who had no road crossings on the way to university. Students with housewife mothers had more odds of injury (OR = 1.32, 95% CI = 1.10 - 1.64, p-value = 0.03) than students with mothers who were employed outside home.

Those who did not always waited for green lights to cross the street had lower odds of reporting an injury (OR= 0.77, 95% CI = 0.61- 1.00, p-value = 0.02). Those who not always looking both ways had two times higher odds of reporting an injury than those who did not (95% CI = 1.50-2.67, p-value < 0.01). Participants who were injured in the road were significantly more likely to believe that RTIs were preventable (OR = 1.52, 95% CI = 1.13-2.06, p-value = 0.004). 

**Attitude Towards RTI Prevention**

In the risk factor analysis for the outcome of “thinking injuries are preventable"(Table 2), presence of a pedestrian injury in the past six months was the only variable significantly associated with increased odds of thinking injuries are preventable, OR 1.53 (95% CI = 1.12-2.08, p-value < 0.01). Factors associated with decreased belief about the preventability of injuries included: students ever playing in the road (OR 0.67 {95% CI = 0.45-0.98, p-value = 0.04}), ever playing in the sidewalk (OR 0.26 {95% CI = 0.14-0.48, p-value < 0.01}), and having family infrequently talk about preventing injuries (OR=0.56, {95% CI = 0.42-0.75, p-value < 0.01}).

**Table:** Table 2:**Relationship between attitude towards RTI prevention and specific associated factors**

Risk Factor	OR for thinking Injuries are preventable	CI	P-value
Ever play in sidewalk	0.255	0.135 - 0.479	<.0001
Ever play in road	0.665	0.453 - 0.975	0.0368
Infrequent education by parents on safe road crossing	0.562	0.424 - 0.745	<.0001
Injury in the past six months	1.526	1.122 - 2.076	<.0001

**Road Behaviors**

Table 3 presents the results of risk factor models comparing people with four, three, and two risk factors to those who only had one. Only variables that were significant at p=0.01 (this p-value is lower than the usual 0.05 to deal with multiple comparisons issue) were left in the final models. Across the three models, education on safe road crossing was found to be protective against having negative road crossing behaviors, but neither the attitudes nor most of the road risk-perceptions were found to be protective against negative road crossing behaviors. However, thinking that it is safe to cross at relatively little traffic was protective against having more road risky behaviors.

**Table:** Table 3:**Relation between road risk perception, education and road crossing behaviors adjusted by gender**

Risk Factor	OR for Having 4 road risk behaviors compared to 1(CI)	OR for having 3 road risk behaviors compared to 1 (CI)	OR for having 2 road risk behaviors compared to 1 (CI)
Road risk perception			
- Perceived safe to cross road at relatively little traffic	0.31 (0.13-0.72)	0.18 (0.08 - 0.42)	0.19 (0.08- 0.44)
Education on safe road crossing			
- Frequent education by parents	0.17( 0.09 - 0.33)	0.24 (0.12-0.46)	0.39 (0.19-0.77)
- Frequent education by lecturers	n/a^*^	0.14 ( 0.06-0.33)	n/a^*^

Not applicable *

**Road Risk Perceptions**

Table 4 presents the outcomes of logistic models including only variables significant at the p=0.01 in the final risk factor model. Road risk-perception analyses were also adjusted for gender. Across all three models, always looking both ways before crossing the road was the most significant protective factor against negative road risk-perceptions.

**Table:** Table 4:**Relation between road crossing behavior, education, and road risk perception adjusted by gender**

Risk Factor	4 perceptions compared to 0	3 perceptions compared to 0	2 perceptions compared to 0	1 perception compared to 0
Always looking both ways before crossing road	0.06 (0.01-0.49)	0.33 (0.15-0.70)	0.55 (0.38-0.81)	n/a^*^
Frequent education by parents on safe road crossing	n/a^*^	n/a^*^	0.58 (0.40-0.84)	n/a^*^

Not applicable *

## Discussion

The study shows a strong association between some participants’ road behaviors and pedestrian injury. There was weak association between their walking and / or playing in the road and the sidewalk and involvement in pedestrian injury. There was also a positive association between road risk-perception and road behaviors, and attitudes toward RTI prevention.

This study showed that the risk of pedestrians' injuries for females approximately to equal males (OR= 0.98 {95% CI = 0.78 - 1.33, p = 0.46}). This is may be due to nearly equal engagement of both males and females in college participation and their free movement in an urban city like Cairo, which place them at an almost equal probability of being exposed to the risk of pedestrian injury. On the contrary, other studies^11,12^showed more males were injured in traffic than females. This can be explained by the fact that males perceive themselves as less susceptible to RTIs, and are more optimistic of their own injury protection and escape skills than females.^11,12^

Our study showed that participants who reside in rural areas were significantly more likely to incur a pedestrian injury than those who reside in urban areas. This can be explained by the unfamiliar, heavily occupied and busy urban traffic environment that rural residents had to deal with. Indeed, most of those who reside in rural areas have to travel on weekly or biweekly basis to and from Cairo to attend their classes, which place them at increased risks of incurring pedestrian-related injuries. Similarly, as a consequence, more pedestrians are exposed to the hazards of crashes when they have to cross one or more roads or travel for a distance that is more than 10 minutes on their way to university.

We also found that students whose mothers were not working outside of the house (i.e. were housewives) were significantly more likely to get injured in the road. This could mean that these mothers were less knowledgeable about pedestrian-related risky behaviors and preventions, therefore less likely to inform their children about these risks. Whereas, working mother are more likely to be exposed to and transfer this information to their children. Hoseth and Rundmo found that people with a higher level of education demanded less transport risk mitigation than individuals with a lower level of education.^13^In a survey on child–parent interaction in relation to road safety education,^14^mothers provided road safety education of some form as well as, reported setting a good example as a safe/careful road user and pointing out unsafe behaviors by others significantly more often than fathers.

This study indicated that those who did not always look both ways before crossing the road (32%) were significantly more likely to get injured than those who did so. A study conducted in Karachi, Pakistan found that only 60% of the pedestrians looked left and right before crossing, 35% of pedestrians caused the traffic to swerve.^15^

Surprisingly, most of the participants (83.3%), who did not always wait for green signals before crossing the road, were less likely to get injured on the street. Since pedestrians' walk signals are absent at most intersections, in Cairo, pedestrians may be accustomed to this and as a result they do not look for them and find them useless. In fact, the drivers and even the traffic police officers often are not effective in enforcing the pedestrians to comply with road signals that are designed for their safety. Moreover drivers, do not honor right-of-way to those crossing the street or pay attention to pedestrian walk signal hence they aggravate the traffic signals' ignorance. Besides, there is no traffic violation citation in the traffic law assigned for pedestrians disobeying the traffic guidelines. A similar behavior of not utilizing road safety features was noted in Rio de Janeiro where safe pedestrian routes (e.g. foot-bridges) are so arduous that pedestrians choose an easier, more hazardous route, i.e. directly crossing the busy street.^16^Meanwhile, several studies reported that lack of knowledge of pedestrians regarding traffic-related rules and regulations does not seem to be the issue; rather, pedestrians want to cross where it is convenient for them and with as little delay as possible.^17-20^Moreover, the enforcement of traffic rules and regulations by traffic police officers is infrequent and they consider it unwarranted.^21^

In the present study, participants' walking and/or playing in the road and the sidewalk, did not show significant association with their risk of pedestrian injury. This may be due to the fact that overwhelming majority of pedestrians injured on public roads are struck while crossing the road, as opposed to while walking on a footpath, walking along the side of a road, or playing on the road.^22^

Our study showed that participants who were injured in the road were significantly more likely to believe that RTIs were preventable. This suggests that exposure to an injury may have had an effect on fostering a positive attitude among these participants, which could be translated into action and adoption of a proper road behavior. Road safety education campaigns can help to build and enforce such positive attitudes, and subsequently improve the road safety behaviors of youth, and as a result help in reducing pedestrian-related injuries.

Our study showed a positive association between students’ road risk-perception and road behaviors, and attitude toward RTI prevention. Deery proposed that higher subjective appraisal of traffic risk increases tendencies to engage in protective traffic behavior.^23^On the contrary, several studies indicate that the cognitive component of traffic risk perceptions (i.e. probability of traffic crashes) and pedestrian behavior is weakly related. Rundmo and Iversen found that perceptions of traffic risk had only weak relationships to reported pedestrian behavior.^12^Furthermore; Wilde’s risk homeostasis theory suggests that different individuals have various levels of acceptable risk, which remain relatively stable over time. Hence, it is possible that counter-measures reduce perceived traffic risk among the individuals, and in order to compensate and increase perceived risk back to the preferred level pedestrian behavior may become more risky.^24^

Our study also showed a positive association between road safety education, attitude to RTI prevention, and road behaviors, and between attitude to RTI prevention and road playing behaviors. Therefore, road safety education deserves to be given much more of a priority in low and middle income countries. Importantly, research has demonstrated that it can be highly effective when some principles of good practice are followed. However, to produce best results, the road safety education programs should be supported by other road safety measures including; driver training, providing safe pedestrian crossing zones and enforcing safe-driver behaviors.^25^

United Kingdom experience shows that using actual streets with real-time traffic have the most potential for road safety training of young children.^26^However, about 20% of schools, surveyed in some low and middle income countries, reported that over-crowded timetable and lack of resources and finance were serious barriers in the teaching road safety to students.^27^

As one of the limitations of this study, there is concern about measurement reliability and validity since the survey relies on self-reported road behavior. However, in our study reasons that may affect reliability of the questionnaire were not present, as there were no ambiguous questions. In addition, sufficient clarifications were provided to all participants about the purpose of the study and the nature of the questions. Regarding the validity, the possible causes of lacking validity, like possibility of the participants to lie or to give answers that one desired, were overcome by Stevenson^28^who found no significant difference between children's reported exposure to the road environment and either observed exposure or exposure reported in pedestrian records. Also self-reports appear to be less accurate for studies that carry social stigma like alcoholism and illegal sexual behaviors. In addition, there were no leading questions and confidentiality was reinforced to allow respondents to give more truthful responses.

Despite such limitations, we have incorporated several aspects of the phenomenon under study such as road behaviors, perceptions of road risks, attitude towards injury prevention, and road safety education by reviewing the literature to ensure content validity.

## Conclusion

Inappropriate youths' road behaviors were significantly associated with pedestrian injury. There was also a positive association between road risk perception and road behaviors, and attitude toward RTI prevention. This suggests that a behavioral approach without modification of the traffic environment (such as provision of crossing signals) might not effectively prevent the occurrence of pedestrian injury in low and middle income countries with poor traffic conditions. Findings of this study, therefore, will be useful in encouraging the Egyptian government to install more traffic signals, and to enforce the regulations made for the safety of pedestrians.
